# Healthy Pregnancies Project: Cluster Randomized Controlled Trial of a Community Intervention to Reduce Tobacco Use among Alaska Native Women

**DOI:** 10.3390/ijerph17249302

**Published:** 2020-12-12

**Authors:** Christi A. Patten, Harry A. Lando, Chris A. Desnoyers, Martha J. Bock, Lucinda Alexie, Paul A. Decker, Christine A. Hughes, Kenneth Resnicow, Linda Burhansstipanov, Rahnia Boyer, Joseph Klejka

**Affiliations:** 1 Department of Psychiatry and Psychology, Mayo Clinic, 200 First St. SW, Rochester, MN, 55905, USA; martha.jane.bock@gmail.com (M.J.B.); hughes.christine@mayo.edu (C.A.H.); 2 School of Public Health, Division of Epidemiology and Community Health, University of Minnesota, 1300 2nd St, Ste 200, Minneapolis, MN 55454, USA; lando001@umn.edu; 3 Yukon-Kuskokwim Health Corporation, 528 Chief Eddie Hoffman Hwy, Bethel, AK 99559, USA; christine_desnoyers@ykhc.org (C.A.D.); lucilla_alexie@ykhc.org (L.A.); rahnia_boyer@ykhc.org (R.B.); joseph_klejka@ykhc.org (J.K.); 4 Department of Health Sciences Research, Mayo Clinic, Harwick 7, 200 First Street SW, Rochester, MN 55905, USA; decker.paul@mayo.edu; 5 School of Public Health, University of Michigan, 109 S. Observatory, 3867 SPH1, Ann Arbor, MI 48109, USA; kresnic@umich.edu; 6 Native American Cancer Initiatives, Inc., 3022 South Nova Road, Pine, CO 80470, USA; lindab@natamcancer.net

**Keywords:** tobacco, pregnancy, community, Alaska Native, women, intervention

## Abstract

Substantial gaps remain in the evidence base for prenatal tobacco use interventions among Indigenous women. Using a cluster randomized controlled trial (RCT), the Healthy Pregnancies Project evaluated a community-level intervention for Alaska Native (AN) women in rural western Alaska. Sixteen villages were randomly assigned to usual care (control, *n* = 8 villages) or usual care plus a community-level intervention delivered by local AN “Native Sisters” (*n* = 8 villages). Outcomes were tobacco use rate at delivery and at 2 and 6 months postpartum, with biochemical confirmation obtained at 6 months. The program had high reach, enrolling 73% of all eligible women screened. Of the 352 participants, 67% used tobacco at baseline. No significant differences emerged between study groups on follow-up in tobacco use rates. More intervention than control participants made a quit attempt at 2 months postpartum (70% vs. 51%, respectively, *p* = 0.012). Participants in both study groups reported the program helped to raise awareness of healthy pregnancies in the study villages. This trial supports the reach of a community-level intervention, but not its efficacy for reducing tobacco use during pregnancy or postpartum. Efforts to sustain early quit attempts appear warranted. The community involvement, and reported impact on raising awareness of the importance of healthy pregnancies, supports the value of the research program in this community.

## 1. Introduction

Worldwide, Indigenous women are more likely to smoke cigarettes and use smokeless tobacco (ST) during pregnancy compared with non-Indigenous women [1]. However, substantial gaps remain in the evidence base for prenatal tobacco use interventions among Indigenous women [1,2]. Among Alaska Native (AN) women residing in the Yukon-Kuskokwim (Y-K) region of western Alaska, prenatal prevalence of smoking is 20%, and 48% use ST [3], primarily Iq’mik, a mixture of tree fungus ash and tobacco leaves [4]. Unlike other Indigenous populations, AN people do not use Iq’mik or other tobacco for ceremonial purposes [5]. A pilot randomized controlled trial (RCT) conducted in this region found that an individual-based intervention delivered at the first prenatal visit had low reach to pregnant women and was not effective for tobacco cessation [6].

In the current Healthy Pregnancies Project, we evaluated a social marketing campaign targeting the entire community, not just pregnant women, to address social norms about tobacco use [7], an approach found to be effective in other contexts for reducing smoking prevalence [8]. In addition to the campaign, we provided individual peer counseling. Both components were delivered by local AN “Native Sisters”/health educators, an approach found to be effective among Indigenous women [9,10]. Using a cluster RCT with village as the unit of assignment, we hypothesized that compared with usual care, the intervention would be associated with a lower tobacco use rate at 6 months postpartum. Secondary outcomes were the tobacco use rates at delivery and at 2 months postpartum.

## 2. Materials and Methods

The trial, registered with the Clinical Trials Registry (NCT02083081), was approved by the Yukon-Kuskokwim Health Corporation (YKHC) Human Studies Committee and Board of Directors and the Alaska Area and Mayo Clinic Institutional Review Boards (IRBs). A Community Advisory Committee guided all aspects of the project. Trial enrollment and data collection occurred between January 2016 and June 2019.

### 2.1. Study Setting

The Y-K Delta region in rural western Alaska comprises 58 federally recognized tribes from 47 village locations ranging from 28 to 1133 persons. There is no road system connecting the villages, which are reached by boat, snow machine, or small plane. The population is mostly of Yup’ik, Cup’ik, or Athabascan ethnicity and of low socioeconomic status. Prenatal care is provided at the Y-K Delta regional hospital, located in Bethel (hub of the village locations), in addition to five Sub-Regional Clinics, and the Anchorage-based Alaska Native Medical Center. At about week 36 of gestation, most Y-K Delta women stay at a Pre-Maternal Home in Bethel or Anchorage before delivery.

### 2.2. Study Design

Sixteen village locations were stratified based on population size (≤600, >600) and randomly assigned to receive the usual care control condition (*n* = 8 villages) or the intervention (*n* = 8 villages) by the study statistician using a computer random number generator. Participants were informed of their village study assignment during the consent process. In-person or phone interview assessments were conducted by research staff at baseline, near the time of delivery, and at 2 and 6 months postpartum. To reduce bias, research staff conducting the assessments did not deliver the intervention, but they were not blinded to the village assignment. Participants received up to USD 125 in gift cards for their time to complete assessments.

### 2.3. Participants

The targeted sample size was 352 (average of 22 pregnant women per village), accounting for estimated 10% attrition, to achieve a final analysis sample of 320 [7]. From previous studies, the tobacco use rate at 6 months postpartum was estimated to be 70% in the control villages and 55% in the intervention villages (estimated odds ratio (OR) 1.91). With 16 villages in total, an average village participant size of 20 (total = 320), and intra-class correlation coefficient = 0.01, the study would have >80% power to detect the hypothesized differences between conditions at 6 months postpartum.

The project was advertised as a “Healthy Pregnancies Study”. Successful recruitment methods used by the research coordinator included enrolling participants at a prenatal care visit in Bethel and utilizing information from the electronic medical record at YKHC regarding pregnancy-based appointments. Research staff also recruited women in person at the Bethel Pre-Maternal Home and in the study villages.

Research staff conducted screenings in person or by phone to determine eligibility based on the following criteria: (1) AN woman; (2) aged ≥18 years; (3) currently pregnant and at ≤36 weeks of gestation at the time of screening; and (4) had access to a working telephone. Both tobacco users and non-users were eligible because an earlier study found that many Y-K Delta women initiate during pregnancy [11], and to enhance the participation/reach of the program with a focus on healthy pregnancies [6,12]. Women were excluded if they resided in a non-study village. Participants provided written informed consent in person or by mail. Next, participants who provided consent completed the baseline assessment in person or by phone. Participants who provided consent and completed the baseline assessment were enrolled. Participants received a gift bag with baby items (e.g., diapers) at enrollment.

### 2.4. Treatments

#### 2.4.1. Usual Care Components Common to All Study Villages

The Clinical Practice Guidelines for pregnant women who smoke recommend provision of pregnancy- and culture-specific written materials on tobacco use risks during pregnancy, and counseling using the 5 As (Ask, Assess, Advise, Assist, and Arrange) [13]. The usual care provided by Health Aides and/or prenatal care providers in this region to all pregnant women (tobacco users and non-users) consisted of providing pregnancy-specific and local adapted written materials on tobacco use risks during pregnancy and minimum cessation counseling (Ask, Advise). Providers referred interested patients to the YKHC clinical tobacco cessation services. The YKHC offered tobacco cessation counseling in Yup’ik or English language in person and/or by phone, as well as nicotine replacement therapy (NRT) and other medication support based on patient needs. Counseling and pharmacotherapy services costs were billed to Medicaid or private insurance or covered with funding from the Indian Health Service. During the study period, the state of Alaska advertised, throughout the region, the availability of free state quitline cessation resources including phone counseling and NRT.

#### 2.4.2. Control Villages

Control villages received the usual care provided as described above. No additional study intervention was provided.

#### 2.4.3. Intervention Villages

In addition to the usual care described above, intervention villages received a locally adapted, community-wide social marketing campaign and, for enrolled pregnant women, individual peer phone counseling. Both components were delivered by 5 local AN “Native Sisters” (NSs) who were Elder (aged >55 years) or, if younger, were in good standing in the community, never or former (≥6 months) tobacco users, and bilingual (English and Yup’ik).

A series of in-person training opportunities for NSs were provided, including training on basic communication skills, with follow-up webinar support as needed. NSs also received training and ongoing support from the YKHC Behavioral Health Program Calricaraq staff. This program addresses historical trauma on health disparities, provides insights on healthy coping mechanisms, and celebrates ancient healthy ways of living. Elder speakers and culture bearers shared in-depth Yup’ik knowledge on how their beliefs, values, and practices applied to the NSs’ intervention from a holistic perspective.

Social marketing campaign: Developed through a participatory and iterative process [12], the campaign messaging focused on healthy pregnancies, e.g., stress reduction and prenatal care. Messaging included a factual, loss-framed approach to describe tobacco use harms for mothers and babies. Campaign media comprised a brochure, posters, promotional items (e.g., baby bibs and hoodies), postcards, and a digital stories DVD. We chose a DVD format because a prior study of 100 AN pregnant women from this region found that 98% reported having access to a DVD player in their home [14] and use of a DVD was feasible in a previous intervention study [6]. Women who were study participants received the DVD and brochure at enrollment in person or by mail. The campaign lasted for about 20 months in each village (June 2017 through January 2019). Postcards describing the NSs’ intervention were mailed to the village post offices for distribution in all resident mailboxes and information was posted on village-specific Facebook pages.

The NSs documented their outreach activities using checklists and field notes. The NSs delivered campaign media in their home village or traveled with research staff to the 8 villages. They distributed the brochures, DVDs, and promotional items; displayed posters; and asked other community members to distribute the materials and/or to be an advocate for the study. They informed Tribal officials of the study activities and had 1-to-1 conversations with community members. Other activities were based on village Tribal officials’ preferences for implementation of the campaign outreach and NSs’ and research staff availability. In 7 of the 8 villages, presentations or group discussions were held where the NSs spoke about the study and their role. In 5 villages, NSs and/or research staff members regularly set up an educational table or booth to discuss the project with community members. Topics addressed during individual and group conversations aligned with Calricaraq holistic teachings and Yup’ik worldview, focusing on overall wellness.

Individual peer counseling: NSs delivered a manualized, phone-based intervention with enrolled pregnant women comprising up to 3 sessions during pregnancy (calls #1, 2, and 3 were delivered at weeks 1, 2, and 4 post-enrollment, respectively), and 3 sessions postpartum (calls #4, 5, and 6 were delivered at weeks 2, 4, and 6 post-delivery, respectively), lasting approximately 10–60 min each. The actual number and timing of sessions depended on when each woman delivered her baby and the NS’ ability to reach the participant. There were challenges in implementing the calls as planned due to NS turnover and participant schedule conflicts or technological issues with cell phones (changed or disconnected numbers and limited minutes). During the initial contact, if the woman was still pregnant, the NS delivered call #1, regardless of when she was enrolled. However, if the woman had delivered, she received the first postpartum call (call #4). Sessions were done in Yup’ik and/or English language, depending on the woman’s preference. NSs were provided with a study phone and maintained a flexible schedule to reach women at various times and days of the week. NSs documented participant completion of each session (of 6 total) and duration.

Session content included evidence-based techniques for being tobacco-free [13], based on the 5 As. Women who currently used tobacco were asked about their readiness to quit. Women who did not use tobacco were encouraged to remain tobacco-free. Evidence-based techniques for quitting tobacco/remaining tobacco-free provided by the NSs were providing support, problem solving, and reinforcement. The NSs encouraged traditional healthy ways of coping with withdrawal symptoms and/or stress such as positive cultural and community activities (e.g., berry picking or walking on the tundra). The NSs also asked the women what additional health topics relevant to pregnancy and the postpartum period they would like to discuss. These included prenatal care, breastfeeding, traditional ways of being healthy, and managing stress. NSs referred interested participants to the YKHC clinical tobacco cessation services, but these services were not provided as part of the study.

Using a checklist, NSs documented each session topic covered in the manual. These checklists were reviewed by local research staff who met weekly with the NSs by phone to discuss and solve issues with implementation. The counseling skill levels of the NSs, assessed during the training sessions, varied greatly. There was significant staff turnover and the research staff attempted to train newly hired NSs directly and provided local shadowing and/or peer training and observation efforts. Because most of the NSs lived and worked in different villages, this latter strategy was available intermittently.

### 2.5. Measures

Reach and retention: Research staff documented the number of screened eligible women who enrolled and participants completing each assessment.

Baseline characteristics: Included age, education, marital status, written and spoken language, gestational age, parity, second-hand smoke exposure [15], and depressive symptoms using the 20-item CES-D (Center for Epidemiologic Studies Depression Scale) [16].

Campaign exposure (intervention villages only): At follow-up assessments, participants reported their level of campaign exposure, using 6 items adapted from prior research [17,18], summed to create a total score. Participants were asked to recall the key campaign message(s) with open-ended responses.

Self-reported tobacco use: At each assessment, participants were asked, “In the past 7 days, have you used any tobacco, even a chew of Iq’mik/blackbull?” (yes/no) [13,19]. If “Yes,” participants were asked about their main product used and any others used (Iq’mik (blackbull), Copenhagen/other chew, cigarettes, e-cigarettes, other, or none).

Biochemical verification: At delivery and at 6 months postpartum, a saliva specimen sample was collected for cotinine analysis [20]. A cotinine kit was mailed to the participant after completing the phone assessment, or research staff collected samples in person; samples were analyzed at Mayo Clinic laboratories. Mailed kits included prepaid postage return labels. A small proportion of all samples (10%) was collected and analyzed at the point of contact using a NicAlert test strip [21].

A procedural error occurred with the delivery assessment only, with many shipping labels excluding the prepaid postage return label. Research staff anecdotally learned that many participants could not pay the postage to return the specimen sample. Of the 313 people completing the delivery assessment, 59% provided a sample (92 in each study group). In contrast, a prior study [6] conducted in this region found a 90% return rate. Thus, at delivery (and at 2 months postpartum), we relied on self-reported tobacco use, with point-prevalence tobacco use defined as self-reported use of any tobacco product during the previous 7 days [19].

At 6 months postpartum, 312 participants completed the assessment, of which 83% provided a saliva specimen sample (131 control, 127 intervention). Mean ± standard deviation delay from self-report to receipt of the sample for mailed kits was 1.5 ± 1.6 months. Despite reminders, research staff learned anecdotally that women either forgot or thought it was too late to return the sample.

At 6 months postpartum, point-prevalence tobacco use was defined as self-reported use of any tobacco product in the past 7 days, and for those reporting abstinence, this was confirmed with a salivary cotinine concentration of ≤20 ng/mL or NicAlert test strip reading of 0 [20,21,22]. Results were classified as tobacco use if (1) the woman reported abstinence but cotinine was missing or elevated (except if using NRT; no participant reported e-cigarette use), or (2) if she reported using tobacco, irrespective of the cotinine result. Concordance of self-reported abstinence and cotinine result was 74% (78% control; 69% intervention).

Quit attempts and non-study tobacco treatment use: At follow-up, current tobacco users reported whether they had tried to quit tobacco since starting the study. Participants reported on utilization of the YKHC clinical cessation services since enrollment, and past 7-day use of NRT or other cessation medications.

Participant feedback: Participants were asked to provide open-ended program feedback.

### 2.6. Statistical Methods

The proportion of eligible women who enrolled, completed each assessment, made a quit attempt, and used tobacco treatment was compared between study groups using generalized estimating equations (GEEs) [23] to account for clustering of outcomes within village. Two research team members independently coded data from participant open-ended responses (inter-rater agreement = 92%); content analysis [24] was used to generate themes. Tobacco use outcomes were defined as self-reported 7-day point-prevalence rate of use of any tobacco/nicotine product. At follow-up at 6 months, self-reported tobacco abstinence was biochemically confirmed with salivary cotinine. The 7-day point-prevalence at each assessment was summarized for each condition and compared between study groups using GEEs in both: (1) an intent-to-treat (ITT) analysis of all 352 randomized participants, with individuals lost to follow-up or with missing data classified as using tobacco, and (2) a per-protocol analysis including only participants who completed the assessment [25] (313 at delivery and 273 at 2 months and 312 at 6-months postpartum). Exploratory analysis was used to examine potential treatment effects separately among baseline tobacco users and non-users. Additional models adjusted for baseline variables that differed significantly between study groups. Among intervention participants, the association of the campaign exposure score, number of NS calls completed, and self-reported tobacco abstinence was examined using GEEs.

For GEE analyses, the appropriate link function was used, e.g., a logit (binomial outcome), cumulative logit (multinomial outcome), or identity (continuous outcome). *p*-values < 0.05 were considered statistically significant.

## 3. Results

### 3.1. Participants

**Figure 1:**
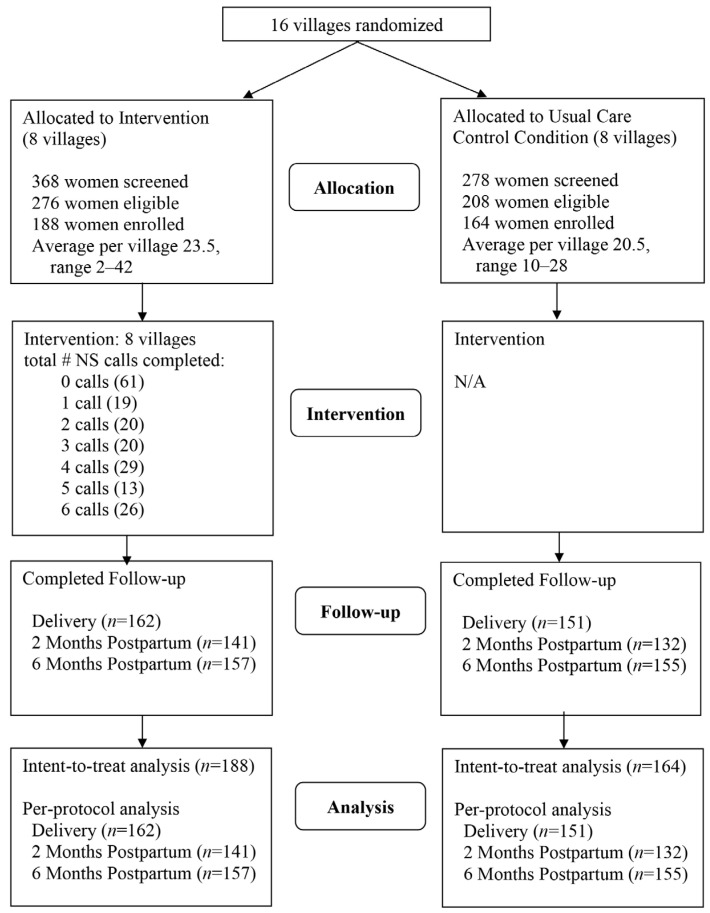
Participant flow.

Figure 1 illustrates the study flow. Participants were *n* = 352 pregnant AN women (188 intervention villages, 164 control villages). The proportion of eligible women enrolled was 73% (352/484). During the recruitment period, the program reached more than half of all pregnant women in the 16 study villages (55%, 352/637). Baseline characteristics were described in detail in a previous report [7]. Briefly, participants’ mean age was 25.8 ± 5.0 years, 83% were married or partnered, 22% reported this pregnancy as their first, and average week of gestation at the time of completing the baseline assessment was 26.8 ± 9.8 (range 6–40). In addition, 44% were employed/working; 25% reported having below high school education; 60% had a high school education; and 16% had some education beyond high school. Current tobacco use was reported by 67% of participants. The main product used was Iq’mik (77%), 18% smoked cigarettes, and 5% used Copenhagen/other ST. As previously reported [7], study groups were comparable on baseline characteristics, except that control participants were more likely than intervention participants to speak and read/write in Yup’ik (69% vs. 53%, *p* = 0.002 and 59% vs. 43%, *p* = 0.003, respectively), report less second-hand smoke exposure (mean # hours/day 1.2 ± 3.7 vs. 1.8 ± 4.0, respectively, *p* = 0.029), and lower mean CES-D score (7.3 ± 6.2 vs. 8.8 ± 6.9, respectively, *p* = 0.044), but were more likely to use Iq’mik (86% vs. 68%, respectively, *p* = 0.006).

Retention was comparable for control and intervention participants at delivery (92% vs. 86%, respectively, *p* = 0.11) and at 2 months postpartum (80.5% vs. 75.0%, *p* = 0.15) (Figure 1). However, control participants were more likely to complete the assessment at 6 months (95% vs. 84%, *p* = 0.02). Reasons for attrition were miscarriage and refusal or unable to contact.

### 3.2. Intervention Implementation

Mean number of calls completed was 2.4 ± 2.2 (range 0–6), with average duration ranging from 22 to 25 min. Completion of ≥1 call during pregnancy was 60% and during postpartum was 50%. Table 1 shows that overall campaign exposure by 6 months postpartum was moderate. The primary barrier reported to viewing the videos was not having access to a DVD player. The key campaign message recalled was “healthy pregnancies.” Others were: “being healthy”, “program to help mother and baby”, and “pregnant women and tobacco use.”

### 3.3. Tobacco Use

No significant study group differences emerged for tobacco use rates at follow-up (Table 2). The primary outcome of biochemically confirmed tobacco use at 6 months postpartum (ITT analysis) was 86% for the intervention and 82% for the control group (*p* = 0.38). Iq’mik was the main product used at follow-up (76% at delivery; 79% at 2 months postpartum; 75% at 6 months postpartum).

Among baseline tobacco users, no intervention effect was detected at follow-up (Table 3). Two additional models adjusted for (1) baseline differences in Yup’ik language, second-hand smoke exposure, and CES-D score; and (2) type of tobacco used at baseline. All variables were not included in one model as there were limited subjects for some of the tobacco types and the models with all variables included failed to converge. Adjusting for these additional variables did not alter the results in either model. Among baseline non-users, significant differences emerged between study groups at 6 months postpartum, with higher rates of biochemically confirmed tobacco use among intervention than control participants. However, this difference was no longer significant after adjusting for baseline covariates. Of the 30 total baseline non-users self-reporting tobacco use at 6 months, 70% smoked cigarettes and 30% reported Iq’mik use. Moreover, 73% (22/30) reported at baseline that they used tobacco before this pregnancy.

Among intervention participants only, a significant association was detected between higher campaign exposure and tobacco abstinence at 6 months postpartum (OR: 1.2; 95% CI: 1.01–1.43; *p* = 0.038). However, no association was detected between tobacco abstinence and number of NS calls completed (OR: 1.02; 95% CI: 0.89–1.18; *p* = 0.76).

At delivery, 63% of participants in each study group reported a quit attempt since enrollment (*p* = 0.89). At 2 months, intervention participants were more likely to report a quit attempt (70% vs. 51%, *p* = 0.012), but no group differences were observed at 6 months (74% vs. 67%, *p* = 0.30).

NRT use (past 7 days) was higher for the intervention than the control group at delivery: 7% vs. 1%, respectively (*p* = 0.022), but not at 2 months (6% vs. 5%) or 6 months postpartum (3% in each study group). No participants reported use of other cessation medications at delivery or at 2 months; one participant (in the control group) indicated their use at 6 months. At delivery, 6% of participants in each study group reported enrollment in the YKHC clinical cessation services. The respective proportions for intervention and control participants at 2 and 6 months was 6% vs. 2% (*p* = 0.14) and 11% vs. 8% (*p* = 0.34).

### 3.4. Participant Feedback

Participants in both study groups noted that the program raised awareness about having a healthy pregnancy. Participants recommended the program be publicized via social media to all women, not just those who are pregnant. Intervention participants recommended holding classes at the Bethel Pre-Maternal Home and providing intervention components via smartphone or social media.

## 4. Discussion

Our community-level intervention was no more effective than usual care for reducing tobacco use during pregnancy or postpartum among AN women. The intervention had some benefits early on, with increased quit attempts at 2 months post-delivery, indicating that additional efforts to support early quit attempts are warranted. A previous pilot study conducted in this region found limited reach of a prenatal cessation intervention, with only 12% of eligible pregnant women enrolled [6]. In contrast, the current trial had good program reach (i.e., 73%), perhaps due to the broader focus on healthy pregnancies and inclusion of pregnant women regardless of their tobacco use status. Participants in both study groups reported that the program increased awareness of healthy pregnancies. As a result of this research, the study team produced videos highlighting the importance of healthy pregnancies that are being disseminated in the region.

The current trial addressed important gaps in prenatal tobacco use interventions among Indigenous women [1,2] and, to our knowledge, is the first to evaluate a community-level intervention. Among pregnant Indigenous smokers, pilot trials found some success with using incentives [26] and providing cessation support from volunteer community workers (“aunties”) [27] or health care providers [28]. However, using biomarker feedback [25] or a locally adapted 5 As intervention [6] was not effective in comparison to usual care. A large RCT conducted in New Zealand (*n* = 297, 40% Maori) found significant effects for a midwife-delivered intervention compared with usual care on smoking cessation during pregnancy but not postpartum [29]. Another RCT enrolling 263 Aboriginal and Torres Strait Islander women (both smokers and non-smokers) found no impact for an intervention, delivered at the first prenatal visit, that engaged significant others for support compared with usual care on the smoking rate at delivery (93% vs. 97%) [30].

The study was advertised as a healthy pregnancies project, not specifically tobacco cessation. Enrolling only tobacco users who wanted to quit may have enhanced the efficacy of the intervention [7]. Barriers related to socio-economic (e.g., educational level) [31] and social-environmental (e.g., high prevalence of tobacco use) [1] factors, as well as the heterogeneity of types of tobacco used, may have also contributed to the lack of intervention effects for tobacco cessation or sustained quit attempts. Furthermore, in our trial, many baseline non-users (21%) reported tobacco use during pregnancy. An earlier report from this region found a higher proportion (75%) of initiation during pregnancy [11]. Women may start to use tobacco when staying at a Pre-Maternal Home due to increased access to Iq’mik and cigarettes for use as a coping strategy in new surroundings (although tobacco use is prohibited on the properties). In univariate analyses, among baseline non-tobacco users, intervention participants were more likely than control participants to report tobacco use at 6 months postpartum. However, in analyses adjusting for Yup’ik language, exposure to second-hand smoke, and depressive symptoms, this difference was no longer significant.

### 4.1. Strengths

This trial evaluated a novel intervention co-designed by community and academic partners and used a rigorous experimental design. The trial was successful in meeting recruitment goals, with high program reach and retention. Tobacco abstinence at 6 months postpartum was biochemically verified.

### 4.2. Limitations

Some aspects of the study population may limit generalizability to other Indigenous pregnant women, such as the heterogeneity in types of tobacco used, the use of Iq’mik, inclusion of non-tobacco users, and restricted geographic location. Only 22% reported this pregnancy as their first, and primiparity is a predictor of successful quitting [31]. Despite the randomization, some baseline characteristics (e.g., Yup’ik language) differed between the control and intervention villages, raising the possibility of selection bias. While overall retention was high, control participants were more likely to complete the 6-month assessment. It is possible that women from the intervention villages may have felt overburdened by calls (i.e., both assessment and NS call attempts).

Our study was not designed to assess the separate effects of the peer counseling and campaign intervention components. However, the results suggest that greater campaign exposure was associated with tobacco abstinence, whereas the individual NS calls were not. It is not clear if the lack of intervention efficacy was due to components of the intervention, the variability of skills of the NSs applying the intervention, and/or its implementation. In this field setting, it was not practical to audiotape the NS calls to assess fidelity. NS call completion was not optimal due to NS turnover and difficulty reaching participants. The level of participant-reported exposure to the campaign outreach by 6 months postpartum was moderate. There were some barriers to campaign delivery, including weather and staff turnover. At 6 months, some women reported not receiving the brochure (8%) or DVD (9%), which may be due to the postal mail service being unreliable depending on the time of year. An additional 39% of study participants reported not watching the DVD, with the most common reason being lack of access to a DVD player. Unfortunately, we did not require having access to a DVD player a study inclusion criteria. Our prior study indicated that a large proportion of pregnant women had access to a DVD player, but advances in technology (e.g., cell phone use) have since occurred that rendered this delivery format impractical for many women.

In an attempt to ensure at least some level of campaign exposure among intervention village residents, postcards were distributed in resident mailboxes and program information was distributed through social media. However, our study was not designed to assess campaign exposure or tobacco use among all residents in intervention villages due to logistical barriers for conducting household surveys.

Perhaps reflective of the complexities of conducting research in remote rural areas, there were barriers to obtaining biochemical verification, and successful but resource-intensive efforts were made by research staff to conduct the 6-month assessment, including travel to the villages. Concordance between self-reported tobacco abstinence and cotinine results was not optimal, but it was comparable to other intervention studies of pregnant smokers [32]. Discrepancies were likely due to the long delays with returning specimen samples via mail. Prior studies among AN pregnant women found very low discrepancy between self-report and biochemical measures assessed simultaneously [25,33]. Moreover, we relied on self-reported tobacco use assessments at delivery and 2 months postpartum [20]. Amount of tobacco used by participants over time was not assessed. Anecdotal reports from the NSs indicated that many women were able to cut down on their tobacco use, even if they did not quit. However, self-reported reductions during pregnancy may have limited long-term significance [34,35].

Interested patients were referred to the YKHC clinical tobacco cessation services and their use was documented, but the study did not promote NRT or use of other medications. We found that, overall, very few women used these services. However, the intervention was associated with a modest increase in NRT use at delivery. Our study did not track participant use of the Alaska state quitline services.

### 4.3. Future Directions

The results suggest several avenues for future research. New methods are available for obtaining biochemical verification in remote settings [36,37]. Conducting online surveys or interviews proximate to collection of the biospecimen sample (e.g., though a phone video call) could enhance response and concordance rates while requiring fewer staff resources. Since the study began, the use of smartphones in the region has become nearly ubiquitous, and thus, digital stories could now be downloaded on an application or delivered via text messages. Social media (e.g., Facebook) is also widely used in the region, which could provide a scalable and sustainable platform for delivering campaign messages [38]. These digital platforms have potential to enhance the reach of, and exposure to, the campaign components. Furthermore, efforts to promote pregnant and postpartum AN women’s use of clinical tobacco cessation services are warranted [25]. While the intervention targeted entire communities, social contextual factors such as support within families may have varied. Anecdotal reports from the NSs indicated that both challenges and successes with the intervention with regards to quitting or reducing tobacco use were tied to family support. Thus, the role of family norms and support merits study.

## 5. Conclusions

This study in rural western Alaska supports the reach of a community-level intervention, but not its efficacy for reducing tobacco use during pregnancy or postpartum. Efforts to support early quit attempts appear warranted. In addition, the community involvement and reported impact of the Healthy Pregnancies Project on raising awareness of the importance of healthy pregnancies support the value of the research program in this community.

## Figures and Tables

**Table 1:** Campaign exposure among intervention village participants ^1^.

	Time Point		
Campaign Component	Delivery (*n* = 156) %	2 Months Postpartum (*n* = 139) %	6 Months Postpartum (*n* = 156) %
1. How much of the brochure did you read?			
None of it	20.5	23.7	13.5
Some	21.8	19.4	22.6
Most	14.7	12.2	12.3
All	34.0	41.7	43.2
Did not receive it	9.0	2.9	8.4
2. How much of the digital stories DVD did you watch?			
None of it	51.3	43.2	39.1
Some	9.6	7.9	9.0
Most	4.5	6.5	5.8
All	25.6	36.0	37.2
Did not receive it	9.0	6.5	9.0
3. Did you share the digital stories DVD, brochure, or promotional items (baby bibs) with family members or anyone else in your community?			
Yes	35.3	52.5	49.7
No	64.7	47.5	50.3
4. Did you talk about what you have learned in this study with anyone else?			
Yes	39.1	56.1	58.7
No	60.9	43.9	41.3
5. Have you seen a poster in your community about the Native Sisters program?			
Yes	62.2	73.4	79.5
No	37.8	26.6	20.5
6. Have others in your village talked about the posters, promotional items (hoodies, baby bibs), or Native Sisters program?			
Yes	23.7	38.8	33.8
No	76.3	61.2	67.2
Total Score (mean ± SD, range) ^2^	3.5 ± 2.1 0–8	4.4 ± 2.3 0–8	4.5 ± 2.3 0–8

^1^ The *n* for each time point reflects the number of women in the intervention group completing the respective assessment. Percentages are based on non-missing data. ^2^ Campaign exposure index, calculated as the sum of the 6 items. Items 1 and 2 were scored as 0 = none of it or did not receive it, 1 = read or watched some, and 2 = read or watched most or almost all. Items 3–6 were scored as 0 = no, 1 = yes. Possible range is 0–8, with higher scores indicating greater campaign exposure.

**Table 2:** Point-prevalence tobacco use rates ^1^ by study group.

Time Point ^2^	Intervention *n* = 188 %, (*n*)	Usual Care Control *n* = 164 % (*n*)	*p*-Value ^1,2^
Baseline	63.8 (120/188)	69.5 (114/164)	0.37
Delivery			
Per protocol	59.3 (96/162)	64.9 (98/151)	0.11
Intent-to-treat	64.9 (122/188)	67.7 (111/164)	0.45
2 months postpartum			
Per protocol	60.3 (85/141)	65.2 (86/132)	0.45
Intent-to-treat	70.2 (132/188)	72.0 (118/164)	0.73
6 months postpartum			
Self-report			
Per protocol	66.9 (105/157)	65.2 (101/155)	0.74
Intent-to-treat	72.3 (136/188)	67.1 (110/164)	0.30
Biochemically confirmed			
Per protocol	82.8 (130/157)	81.3 (126/155)	0.73
Intent-to-treat	85.6 (161/188)	82.3 (135/164)	0.38

^1^ Study group differences at each follow-up assessment were analyzed using generalized estimating equations (GEEs) to account for the cluster randomized study design (village). ^2^ An additional model adjusted for baseline differences between study groups; adjusting for these variables did not alter the results.

**Table 3:** Proportion of women using tobacco at follow-up stratified by baseline tobacco use status.

	Baseline Tobacco Users			Baseline Non-Tobacco Users		
Time Point	Intervention Group *n* = 120 %, (*n*)	Usual Care Control Group *n* = 114 % (*n*)	*p*-Value ^1^	Intervention Group *n* = 68 %, (*n*)	Usual Care Control Group *n* = 50 % (*n*)	*p*-Value ^1,2^
Delivery						
Per protocol	78.9 (82/104)	84.9 (90/106)	0.15	24.1 (14/58)	17.8 (8/45)	0.51 (0.40)
Intent-to-treat	81.7 (98/120)	86.0 (98/114)	0.30	35.3 (24/68)	26.0 (13/50)	0.36 (0.56)
2 months postpartum						
Per protocol	81.4 (70/86)	88.0 (81/92)	0.24	27.3 (15/55)	12.5 (5/40)	0.075 (0.05)
Intent-to-treat	86.7 (104/120)	90.4 (103/114)	0.38	41.2 (28/68)	30.0 (15/50)	0.20 (0.45)
6 months postpartum						
Self-report						
Per protocol	83.7 (82/98)	84.7 (94/111)	0.88	39.0 (23/59)	15.9 (7/44)	0.015 (0.04)
Intent-to-treat	86.7 (104/120)	85.1 (97/114)	0.74	47.1 (32/68)	26.0 (13/50)	0.040 (0.09)
Biochemically confirmed						
Per protocol	94.9 (93/98)	97.3 (108/111)	0.35	62.7 (37/59)	40.9 (18/44)	0.014 (0.18)
Intent-to-treat	95.8 (115/120)	97.4 (111/114)	0.51	67.7 (46/68)	48.0 (24/50)	0.043 (0.19)

^1^ Study group differences at delivery and at 2 and 6 months postpartum were analyzed using generalized estimating equations (GEEs) to account for the cluster randomized study design (village). ^2^ *p*-value in parentheses adjusts for baseline differences found to differ between study groups.
